# Mitochondrial Chaperones in the Brain: Safeguarding Brain Health and Metabolism?

**DOI:** 10.3389/fendo.2018.00196

**Published:** 2018-04-26

**Authors:** José Pedro Castro, Kristina Wardelmann, Tilman Grune, André Kleinridders

**Affiliations:** ^1^Department of Molecular Toxicology, German Institute of Human Nutrition (DIfE), Potsdam-Rehbruecke, Germany; ^2^German Center for Diabetes Research (DZD), München-Neuherberg, Germany; ^3^Central Regulation of Metabolism, German Institute of Human Nutrition (DIfE), Potsdam-Rehbruecke, Germany; ^4^German Center for Cardiovascular Research (DZHK), Berlin, Germany; ^5^Institute of Nutritional Science, University of Potsdam, Nuthetal, Germany

**Keywords:** insulin signaling, brain, chaperones, mitochondria homeostasis, mitochondrial dysfunction, neurodegeneration

## Abstract

The brain orchestrates organ function and regulates whole body metabolism by the concerted action of neurons and glia cells in the central nervous system. To do so, the brain has tremendously high energy consumption and relies mainly on glucose utilization and mitochondrial function in order to exert its function. As a consequence of high rate metabolism, mitochondria in the brain accumulate errors over time, such as mitochondrial DNA (mtDNA) mutations, reactive oxygen species, and misfolded and aggregated proteins. Thus, mitochondria need to employ specific mechanisms to avoid or ameliorate the rise of damaged proteins that contribute to aberrant mitochondrial function and oxidative stress. To maintain mitochondria homeostasis (mitostasis), cells evolved molecular chaperones that shuttle, refold, or in coordination with proteolytic systems, help to maintain a low steady-state level of misfolded/aggregated proteins. Their importance is exemplified by the occurrence of various brain diseases which exhibit reduced action of chaperones. Chaperone loss (expression and/or function) has been observed during aging, metabolic diseases such as type 2 diabetes and in neurodegenerative diseases such as Alzheimer’s (AD), Parkinson’s (PD) or even Huntington’s (HD) diseases, where the accumulation of damage proteins is evidenced. Within this perspective, we propose that proper brain function is maintained by the joint action of mitochondrial chaperones to ensure and maintain mitostasis contributing to brain health, and that upon failure, alter brain function which can cause metabolic diseases.

## Mitochondria Impact Brain Function

Though the brain holds about 2% of total body mass, it consumes about 20% of total body energy. This high energy demand is due to constant brain action, even when at rest (1). The main energy supply represents glucose and liver-derived ketone bodies during fasting. Mitochondria are able to metabolize the end product of glycolysis pyruvate, as well as ketone bodies, to generate ATP. Mitochondria regulate oxidative phosphorylation, redox state, oxidative stress, intracellular signaling, ion homeostasis, and thus, provide the energy and ion milieu for proper neuronal action and excitability. In addition, mitochondria affect protein acetylation by providing the necessary acetyl-CoA moieties for histone acetylation, thereby affecting chromatin structure and gene transcription of stress responsive genes (2, 3). Therefore, mitochondria occupy a unique position in regulating brain function and metabolism on various levels. Not surprisingly, most defects of mitochondria are linked to neurodegenerative and metabolic diseases (4–6).

The brain regulates metabolism by controlling, e.g., food intake, satiety, and hepatic glucose production, and thus aberrant mitochondria in the brain are connected to insulin resistance (IR) and to metabolic diseases such as metabolic syndrome and type 2 diabetes (T2D) (7–10) as well as aging (11). Conversely, many aspects of brain alterations seen during aging are also observed in metabolic diseases pointing to common origins and linked to mitochondrial dysfunction (12, 13).

To facilitate brain metabolism, metabolites have to enter the brain *via* blood vessels, taken up mostly by glia cells, which reside in close proximity to blood vessels and transferred to neurons (14). As each brain cell population differs regarding their function and cellular components, also mitochondrial function differs between cell populations (15, 16). Even more complex is the scenario within neurons, where mitochondria at the soma are important for generating energy for cellular survival, and at the synapses are crucial for providing ATP for vesicle release and neurotransmitter uptake (17). A reduction in neuronal ATP levels deteriorates normal firing rate causing abnormal brain function (18, 19). Glia release ATP, which reduces neuronal firing rate, indicating that alterations in mitochondrial generated ATP in glia, can also change neuronal activity and impact brain function (20). Mitochondrial dysfunction and reduced ATP production in glia cells do not cause immediate cellular degeneration but affects neuronal homeostasis due to an intensified neuron-glia crosstalk and it promotes neurodegeneration (21, 22). Increased reactive oxidative species (ROS), a byproduct of mitochondrial respiration, can also propagate an inflammatory response, abnormal neuronal firing and associates with IR, metabolic diseases and aging (23). ROS is especially detrimental to neurons because of their low antioxidant capacity, such as dopaminergic neurons of the *substantia nigra*, which degenerate in Parkinson’s disease (24).

Mitochondria are not static; they fuse and split to generate a dynamic network. Alterations of mitochondrial dynamics affect neuronal action and can cause neurodegeneration and obesity (8, 9). Increased mitochondrial fusion in dopaminergic neurons causes axonal loss and contributes to cell death and neurodegeneration (25). In addition, deficiency of prohibitins, which induces mitochondrial fragmentation, leads to reduced ATP production, abnormal mitochondrial morphology, protein aggregation, and causes neuronal death (26), indicating the importance of proper mitochondrial dynamics for neuronal health. Deteriorated mitochondria will be eliminated and recycled by a cytoprotective pathway called mitophagy, which depends also on proper mitochondrial dynamics. Aberrant mitophagy leads to increased oxidative stress and is linked to neurodegenerative diseases such as AD, PD, and HD (27–29). The Mitochondrial Unfolded Protein Response (UPRmt) can regulate this process, where the accumulation of misfolded mitochondrial proteins induces PARK2/Parkin-mediated mitophagy (30). UPRmt represents a signaling pathway where the abundance of misfolded mitochondrial proteins causes a nuclear signal to re-establish protein homeostasis by inducing mitochondrial chaperones and proteases to re-instate protein homeostasis within mitochondria (31). Alterations of the mitochondrial proteome due to misfolding of proteins or protein aggregation decreases mitochondrial activity, causes oxidative stress and neurodegenerative diseases (32–35). Accordingly, proper mitochondrial function and regulation of its stress response has been shown to associate with or even promote longevity (36, 37). Thus, mitochondrial chaperones occupy a pivotal role in regulating mitochondrial health and brain function.

## Hypothesis

In recent years, several studies have shown the importance of functional mitochondria in regulating brain metabolism. Here, we go one step further and postulate that the disruption of the mitochondrial chaperone network in the brain, which controls mitochondrial proteostasis and function, is responsible for IR in the brain and concomitant altered metabolism. This alteration reduces brain function and neuron plasticity resulting in changes in whole body metabolism and increasing the risk for metabolic complications.

Our assumptions rely on studies showing many of the features. We could show that type 2 diabetic mice exhibit a reduction of the heat shock protein 60 (Hsp60) mRNA in the hypothalamus, a mitochondrial chaperone which is part of the UPRmt. A reduction of Hsp60 is sufficient to induce hypothalamic IR (7). Mice deficient for the mitochondrial protease ClpP, a protease involved in UPRmt, exhibit increased food intake (38), a phenotype of deficient brain insulin action showing that altered UPRmt directly affects brain function and brain insulin-regulated behavior (39). Interestingly, while deficiency of ClpP causes hyperphagia, these mice are paradoxically protected against diet-induced IR, exhibiting increased insulin sensitivity in white adipose tissue with increased expression of Hsp60 and Hsp10, strengthening the hypothesis that increased mitochondrial chaperones expression is beneficial for insulin sensitivity (38). Another recent paper showed that ClpP deficiency decreases adaptive thermogenesis (40), which has been also observed in mice deficient for brain insulin signaling (41). Though it is unclear whether ClpP deficiency alters mitochondrial chaperone expression in the brain, the effect that alterations of the mitochondrial proteome in the brain causes brain insulin resistant phenotypes support our hypothesis that ensuring proper mitochondria homeostasis (mitostasis) in the brain supports brain health and proper metabolism.

Furthermore, it has been shown that there is an intricate connection between mitochondrial dynamics and the UPRmt, as UPRmt induces the expression of genes responsible for mitochondrial dynamics (42). Functional mitochondrial dynamics, which are regulated by mitochondrial chaperones, regulate Agouti-related peptide neuronal activity to avoid diet-induced obesity (8). Adding to this, under high fat diet (HFD) dynamin-related protein 1 (DRP1), another crucial protein for mitochondrial dynamics, increases mitochondrial fission in the *Dorsal Vagal* Complex, and impairs insulin action. Also, DRP1 alone was able to induce IR in healthy rodents and inhibiting DRP1 was sufficient to reverse HFD-induced IR (43). Consistent with these results, diet-induced obesity increases fission and alters the contacts between mitochondria and the ER showing the importance of a proper crosstalk between organelles in order to maintain metabolic regulation. Moreover, the same study showed that a specific-POMC neurons Mitofusin-2 ablation resulted in the loss of contact between the mitochondria and ER leading to leptin resistance and energy imbalance (9), both clearly signs of metabolic dysregulation. Exercise as an intervention improves mitochondrial dynamics, increases insulin sensitivity, and is able to modulate the Hsp response in the brain by increasing the levels of many of the Hsps such as Hsp60. They also found that this effect was even stronger in non-diabetic mice able to produce insulin (44). Also noteworthy, is a study where a combination of mouse population genetics and RNAi in *C. elegans* was used to identify mitochondrial ribosomal protein S5 and other mitochondrial ribosomal proteins (MRPs) as regulators of metabolism and longevity. Remarkably, when the authors performed a MRP knockdown mitonuclear protein imbalance was triggered, mitochondrial respiration was reduced but this activated UPRmt, and an increase life span was seen. Moreover, compounds known to extend life span such as spermidine or resveratrol are able to induce mitonuclear protein imbalance as well as trigger the UPRmt (45).

Furthermore, key genes of the UPRmt are regulated by insulin in hypothalamic neurons (own observation). Fitting to this, key players of UPRmt, such as Hsp60 are decreased in insulin-resistant mice brains as well as mouse models deficient for insulin signaling highlighting the importance of functional brain-specific insulin signaling for the regulation of the UPRmt [own observation and (7)]. To further strengthen this point, reduced levels of Hsp60 were found in brain samples of type 2 diabetic patients, which caused mitochondrial dysfunction and brain IR (7).

**Figure 1:**
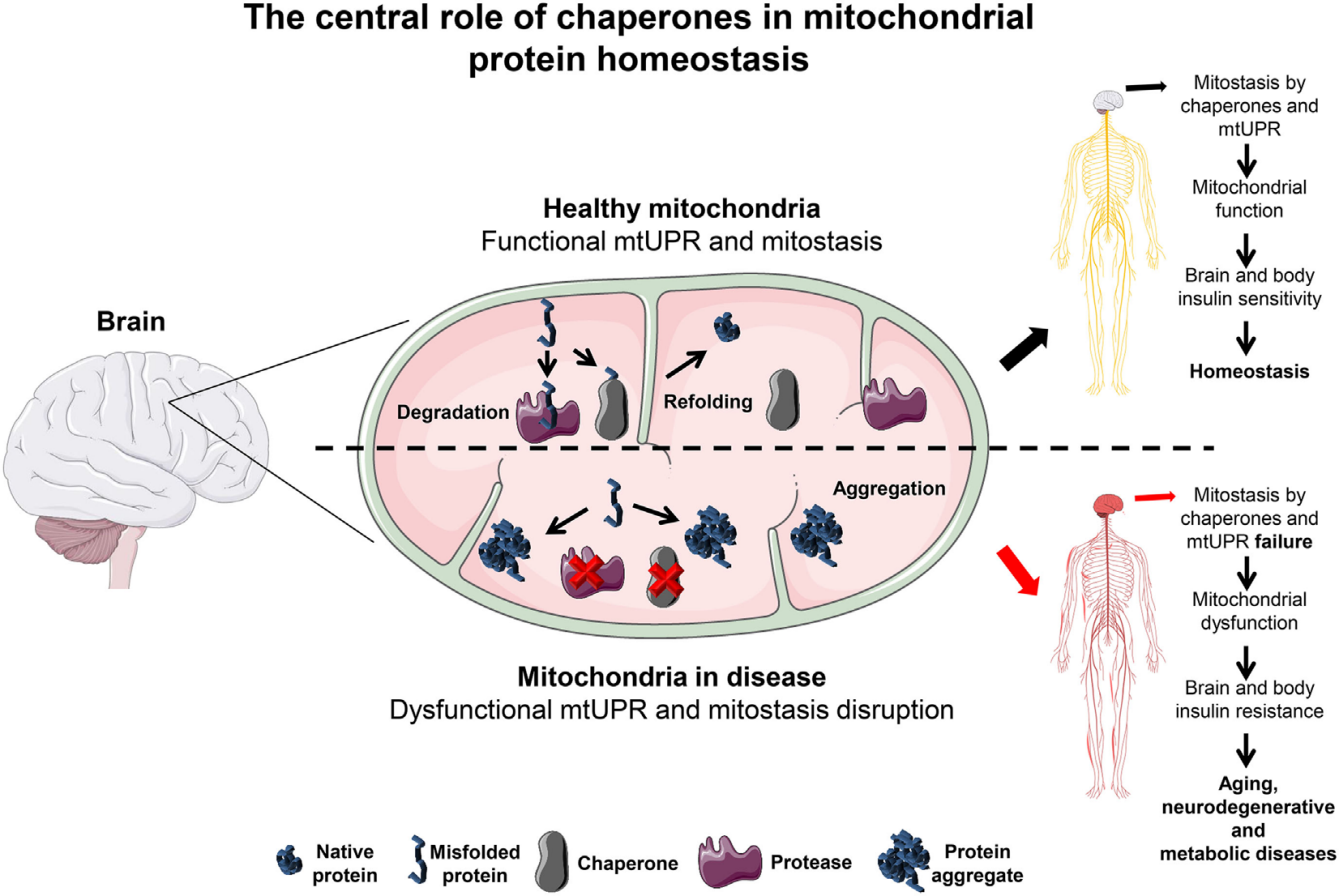
The central role of chaperones in mitochondrial protein homeostasis. In the brain, mitochondria are kept under optimal functional conditions by employing two major mitochondria homeostasis (mitostasis) processes, (re)folding and degradation. Under tight regulation and concerted action these processes avoid the accumulation of different types of damage such as ROS, mitochondrial DNA mutations or misfolded/aggregated proteins known to promote mitochondrial dysfunction. Moreover, a mild increase in the level of misfolded proteins triggers the UPRmt response that quickly helps to establish mitostasis. In the brain, under these conditions, chaperones promote mitochondrial function keeping the brain and whole body in an insulin sensitive state. In contrast, if refolding or degradation processes become impaired due to the loss of mitochondrial chaperones expression or activity this results in misfolded and aggregated protein accumulation. This detrimental state leads to oxidative stress that can affect mitochondrial function and thus brain metabolism. A likely consequence is the resistance to insulin not only initially in the brain but also to peripheral tissues later on, abrogating whole body metabolic homeostasis. This metabolic shift is a likely percursor for aging, neurodegenerative and metabolic diseases progression, and establishment. Servier Medical Art by Servier is licensed under a Creative Commons Attribution 3.0 Unported License.

Taking all into account, we here propose that the disruption of the mitochaperone network induces mitochondrial dysfunction in the brain. This process can trigger brain IR, which alters food intake, induces obesity, and thus affects overall metabolism (41) (Figure 1). The following chapters are intended to provide an overview on the current knowledge and how it contributed to formulate our hypothesis. We here provide evidence on how chaperones are crucial players for mitostasis and brain insulin sensitivity and on how their impairment leads to mitochondrial dysfunction and may link neurodegenerative and metabolic diseases.

## Chaperones as Mitostasis Keepers

### Sources of Mitochondrial Protein Misfolding

Upon mitochondrial dysfunction, it is not surprising that brain cells enter an energy and function deficit. The reasons that promote mitochondrial dysfunction over time or under metabolic dysregulation are now starting to be unraveled.

Evidence suggests that the main reasons may be the exacerbated ROS production and/or mitochondrial DNA (mtDNA) mutations that affect directly or indirectly the proteome of the mitochondria (hereafter referred as mitoproteome). Nowadays, it is clear that mitochondrial dysfunction is a hallmark of tissue aging and the brain is clearly to be included (46, 47); however, it remains unclear if during aging mitochondrial dysfunction is a cause or consequence that triggers the process (48). Regardless of which, mitochondria impact brain aging and metabolism. In fact, mitochondrial ROS leakage was proposed in the 1980s and later updated as the cause of aging itself, as the “mitochondrial free radical theory of aging” postulates (49). All tissues, although with different severity, seem to be affected. The brain is no exception in how the effects of mitochondrial dysfunctional on organ function is concerned. In fact, functional neuroimaging studies have shown that hypometabolism and mitochondrial dysfunction are early hallmarks of age-related modifications during brain aging and during the progression of age-related brain diseases (50–52).

In the brain, mitochondria are widely believed to be the main cellular source for the production of ROS, especially by complex I and III electron leakage, which generates superoxide anion after an incomplete water molecule reduction (53). During aging the leakage is intensified and the risk for molecule damage increases. For example, due to its localization, mtDNA is one of the primary targets which may lead to mutations and generate faulty electron transport chain (ETC) proteins contributing to cellular dysfunction. Moreover, the mitoproteome might be directly affected by ROS attack, which often results in protein carbonylation (54). This type of oxidative modification is an irreversible non-enzymatic event, which can result in protein unfolding, exposure of the usually concealed hydrophobic core and, therefore, protein aggregation (54).

Regarding ROS-independent mtDNA alterations, mutations and deletions caused by rearrangements in the mitochondria accumulate over time and account, at least in part, for the age-associated decline (55). mtDNA mutations have been shown to occur in the brain’s cortex and *substantia nigra* during aging and Parkinson’s disease development (56). However, although ROS- and mtDNA mutations-mediated protein misfolding are undoubtedly important, there are many other factors that contribute to protein misfolding such as errors in mitochondrial or cytosolic protein translation and also disrupted stoichiometry in ETC complex assembly. Remarkably, the simple fact that most of the mitochondrial proteins are nuclear-encoded that need to be translated in the cytoplasm and imported into the mitochondria in an unfolded state reinforces the necessity of a specialized group of nuclear encoded-proteins named molecular chaperones (57).

### Chaperone–Dependent Mitostasis

Molecular chaperones are crucial to assure mitochondrial proteostasis (hereafter referred as mitostasis). They can be grouped into Hsp70, Hsp90, DNAJ/Hsp40, chaperonin/Hsp60, and small heat shock protein (sHsp) families (58). The mechanism of action of each chaperone is complex and may result from individual chaperone action or in combination with co-chaperones that regulate their interaction and activity with client proteins (59). In fact, co-chaperones play a key role as well, shown for example by mtHsp70 co-chaperones such as HSC20, which enables mtHsp90 peptide binding activity (60) and DNAJA3 (also named TID1) that helps to prevent complex I aggregation and takes part in mtDNA maintenance (61). Moreover, another chaperone named Mdj1p has been shown to be involved not only in protein folding but also in mitochondrial biogenesis showing that the same chaperone can have different mitochondrial tasks. However, whether a homolog protein with similar functions is also present in mammalian cells still needs validation (62).

**Figure 2:**
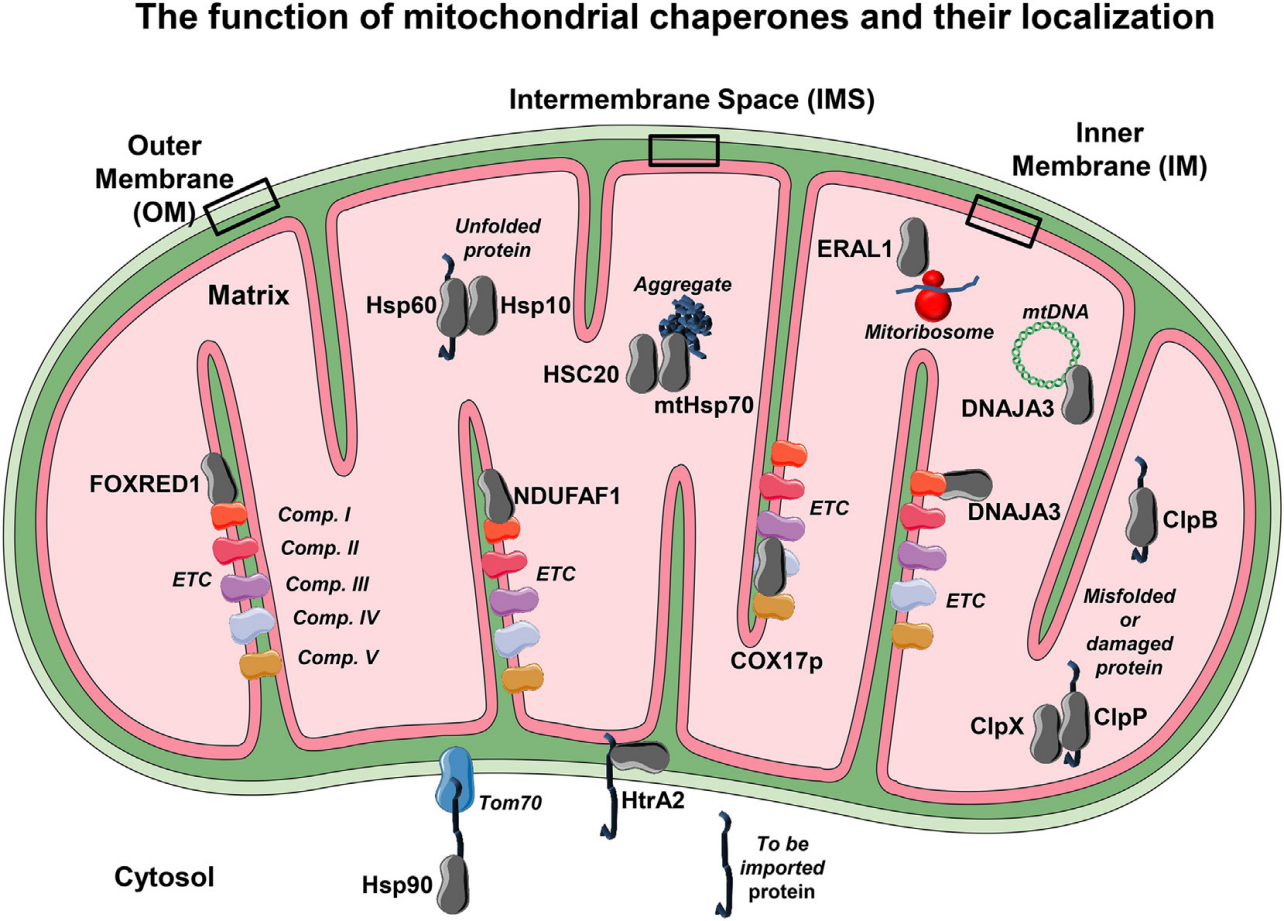
The mitochondrial chaperones localization. Due to the elevated mitochondrial activity in each compartment, it becomes necessary to employ mitochondrial chaperones to ensure proper function. The localization of several chaperones and co-chaperones and their association with several of their targets is shown. Each chaperone or co-chaperone is placed in different mitochondrial compartments such as outer membrane (OM), inner membrane (IM), intermembrane space (IMS), and the matrix according to their main function. From the cytosol to the OM, Hsp90 assists the delivery of *to-be-imported* proteins to the mitochondrial translocase Tom70. Within the IMS, several chaperones such as HtrA2 allow unfolded proteins to translocate from OM to the IM and avoid degradation. On the IM reside the electron transport chain (ETC) proteins, so there are several IM chaperones that have the ability to bind and guarantee proper folding and function to ETC complexes such as NDUFAF1 and *FAD-dependent oxidoreductase-containing domain 1*, which bind to *NADH:ubiquinone oxidoreductase* (complex I). Moreover, COX17p binds to cytochrome C oxidase (complex IV) and helps with its assembly. It is however on the matrix that most of the mitochondrial chaperones or co-chaperones are localized. For example, the chaperone heat shock protein 60 cooperates with its co-chaperone Hsp10 in order to promote correct protein folding. The mtHsp70 and its co-chaperone HSC20 help to promote protein folding and avoid protein aggregation, a detrimental feature that leads to mitochondrial dysfunction. Other chaperones exhibit more than one task. This is the case of DNAJA3, a chaperone that avoids complex I aggregation but is also able to assist mitochondrial DNA maintenance. Other crucial processes such as mitochondrial protein synthesis rely on a proper mitoribosome assembly, a task that is employed by the RNA chaperone ERAL1. In order to keep mitochondria homeostasis—besides correct (re)folding and synthesis—protein degradation is also mandatory. To this effect, mitochondria have proteases such ClpP and ClpX that play a role in degrading no longer required, misfolded, or damaged proteins, and ClpB mainly as a chaperonin and a disaggregase. The conformational display of each protein or complex and binding layout is merely illustrative.

The nature of each specific interaction between these formed complexes can result in a wide range of outcomes for the client protein such as folding, disaggregation, degradation, or trafficking within the cell (59) (Figure 2).

Within the mitochondria, there are four different compartments where protein folding and assembly take place: the outer (OM) and inner membrane (IM), the intermembrane space (IMS), and the matrix. During stress, each compartment must be scanned in order to avoid misfolded protein accumulation (63). In order to maintain mitostasis, in the different compartments, different molecular chaperones must take action accordingly.

#### In the OM

For example, for the OM, evidence shows that Hsp70 and Hsp90 are necessary for proper protein folding. This is not surprising since the OM is in close contact with the cytosol and as mentioned above, most of the mitoproteome needs to be imported into the mitochondria in an unfolded state, increasing the risk for aggregation. These chaperones hold the *to-be-imported* proteins in an unfolded (but soluble) state until they are delivered to the mitochondrial OM import translocase Tom 70 (64). Interestingly, Hsp70 and Hsp90 have mitochondrial homologs named Mortalin (mtHsp70) and tumor necrosis factor receptor-associated protein 1 (TRAP1), respectively (65).

Moreover, mitochondrial dysfunction can induce cytosolic chaperone expression. One striking example shows that alteration in mitochondrial ceramide biosynthesis pathway triggers, either by genetic or pharmacological inhibition of carnitine palmitoyltransferase activity, the induction of chaperones (66). Another example shows that silencing of mtHsp70 in mammalian models of polyglutamine aggregates, typically seen in Huntington’s disease, leads to the activation of UPRmt, resulting in restoration of cellular proteostasis, diminishment of protein aggregates and finally amelioration of mitochondrial damage (66).

The UPRmt can be triggered not only by loss of mitostasis but also when the nuclear- encoded mitochondrial protein expression is not balanced which generates stress to the cell (67, 68). Interestingly, stress responses have the ability to restore proteostasis intra- and inter-cellularly. A striking example comes from the nematode *C. elegans*, in which genetic disturbance of the thermosensory neurons modulates the whole organism’s response to acute or chronic stress (69, 70).

Besides chaperones, mitostasis is also maintained by damage removal pathways. This includes both the degradation of whole mitochondria by mitophagy and a set of intramitochondrial proteases. Both systems respond to stress and act in a concerted action (71). Mitostasis is also achieved by regulated protein turnover mainly performed by Lon protease (LonP) or ClpP and its partner chaperone ClpX. In fact, their function is so important for mitostasis that deficiency of ClpP causes upregulation of mitochondrial chaperones and alters tissue-specific metabolism (38, 40). Deficiency of another protease ClpB leads to progressive brain atrophy *in vivo* and to inhibition of mitochondrial network restoration *in vitro* highlighting the importance of proper mitostasis (Table 1). Again this process seems to be regulated specifically by a chaperone, the cytosolic Hsp90 which has the ability to modulate mitochondrial protein turnover. Thus, the inhibition of Hsp90 leads to a decrease in the degradation of mono- and polyubiquitinated ATP5o, a subunit of ATP synthase, and promotes alterations in the mitochondria morphology which can lead to mitostasis breakdown (72, 73). Moreover, either by mutations in Parkin, an E3 ubiquitin ligase, or by the loss of function of PINK1 (a mitochondrial surface protein target for Parkin), a decrease in neuronal mitophagy is observed and known to directly promote the progression of PD (74, 75). Adding to this, PD patients display augmented IR (76, 77). Taken together, the evidence implies that mitostasis is necessary to maintain insulin sensitivity in the brain.

**Table 1:** Chaperones and mitochondrial dysregulation in neurodegenerative and metabolic disease(s).

Chaperone/Chaperonin	Function	Disease (CNS)	Deficiency/Knockdown	Reference
Hsp10	Co-chaperone of heat shock protein 60 (Hsp60); folding of mitochondrial proteins	Neurological and developmental disorder	*In vitro* KO: cell death	(78–80)
Hsp60	Folding of mitochondrial proteins	SPG13; MitCHAP60 disease	*In vitro* KO: cell death, *In vivo* KO: embryonic lethality	(7, 33, 34)
mtHsp70	Folding of mitochondrial proteins	Contributes to PD pathology	*In vivo* KO: embryonic lethality, *In vitro* KD: induced mitochondrial proteolytic stress	(81–83)
Tumor necrosis factor receptor-associated protein 1	Protection against oxidative stress and mitochondrial cell death, regulation of mitophagy and mitochondrial dynamics	Congenital abnormalities of the kidney and urinary tract; ischemic damage; increases PD pathology when expression is reduced	*In vivo* KO: exhibit reduction in age-associated pathologies; *In vitro* KO: Increased mitochondrial respiration and fatty acid oxidation	(84–90)
ERAL1	RNA chaperone; formation of the 28 S small mitoribosomal subunit	Perrault Syndrome	*In vitro* KD: induces apoptosis	(91–93)
HSC20	Iron-sulfur cluster co-chaperone; regulation of the ATPase and peptide-binding activity of mtHsp70	Not known	*In vitro* KD: reduces complex II assembly	(60)
DNAJA3	Co-chaperone of mtHsp70; stimulation of the ATPase activity of mtHsp70, prevention of complex I aggregation	Implicated in PD	*In vivo* KO: embryonic lethality; *In vitro* KD: induces mitochondrial fragmentation	(61, 94)
CLPB	Mitochondrial AAA ATPase chaperonin, disaggregase	Progressive brain atrophy, Autosomal-recessive mitochondrial disorder	*In vitro* KO: confers thermotolerance to mitochondria, inhibits restoration of mitochondrial network	(95–99)
CLPX	Remodels the conformations of aggregates, partner of ClpP (protease); activation of heme biosynthesis	Not known	Not known	(100, 101)
COX17p	Copper chaperone; assembly of cytochrome C oxidase	Not known	*In vivo* KO: embryonic lethality; mutations: COX deficiency	(102, 103)

*Several crucial mitochondrial chaperones and co-chaperones and their main functions are shown. Upon their failure either by the loss of expression or activity results in manifold diseases of the central nervous system (CNS). Consequences of deficiency or knockdown in experimental set ups and its consequences are also displayed*.

#### In the IM

Proteins of the ETC reside in the IM, which is therefore a critical mitochondrial sub-localization requiring exquisite protein quality control. In fact, the assembly of complex I seems to be highly coordinated by IM chaperones such as *NADH:ubiquinone oxidoreductase complex assembly factor 1* (NDUFAF1) (104). In addition, the chaperone *FAD-dependent oxidoreductase-containing domain 1* (FOXRED1) seems to be crucial for the correct assembly of complex I and II as its mutation results in cell-type-specific assembly defects of these complexes (105). Interestingly, defects in complex I are overrepresented in PD and aging (106, 107) and can contribute to mitochondrial-mediated oxidative stress, which is highly involved in the establishment of IR (108–110), reinforcing the point that mitostasis is crucial to neuronal health.

#### In the IMS

In the IMS, there are several small proteins that can partially act as chaperones allowing unfolded proteins to translocate from OM to the IM and avoid off-pathways such as degradation (111). One of these proteins is the *high temperature requirement A2* (HtrA2 or Omi), which possesses the common chaperone’s ability to bind hydrophobic residues by the use of its C-terminal PZD domain (112) and exhibits proteolytic activity (113). In fact, targeted deletion of HtrA2 gene, Prss25, leads to the loss of a population of neurons in the *corpus striatum*, resulting in a neurodegenerative disorder with parkinsonian features (113).

#### In the Matrix

The matrix hosts several critical cellular processes such as protein synthesis, tricarboxylic acid cycle, or fatty acid oxidation, and it becomes clear that molecular overcrowding is likely to happen. To ensure mitostasis at the matrix level, cells employ mainly two chaperone systems the mtHsp70 and the Hsp60/10 complexes (114–117). In recent years, the importance of Hsp60 as a mitostasis keeper has been evidenced and reviewed extensively. Hsp60 has been shown to be crucial in delaying metabolic complications and age-related diseases. Remarkably, long lived mammals and birds express higher levels of Hsps (118), clearly showing their role in life maintenance. By displaying an essential role on maturation and maintenance of mitoproteome, it becomes intimately associated with energy production and thus regulation of metabolism. For example, a key process for mitochondrial homeostasis is the regulation of its protein synthesis which depends, among several other critical steps, on mitochondrial ribosomal formation. This is achieved by the RNA chaperone ERAL1, which is associated with the formation of the 28S small mitochondrial ribosome, and it plays such an important role that its depletion results in mitochondrial dysfunction and growth retardation (91).

As a conclusion and to support the view on the dependence of molecular chaperones on ensuring mitostasis, their dysregulation is linked to protein aggregation seen in neurodegenerative diseases (119) and aging (57, 120). In metabolic complications such as diabetes or IR (121, 122), altered mitostasis can be a consequence of chaperone machinery dysregulation (7).

Evidence from Carvalho et al. (123) showed “metabolic alterations induced by sucrose intake and Alzheimer’s disease promote similar brain mitochondrial abnormalities,” in which the authors described many resembling features such changes in the respiratory chain and oxidative phosphorylation, dysregulation of calcium content, and morphological abnormalities. Strikingly, sucrose-treated wild-type mice presented a significant increase in amyloid β (Aβ) protein levels, which is a very well established hallmark of AD and also IR. Moreover, another study shows that the loss of the co-chaperone DNAJC3 leads to T2D and massive neurodegeneration (124). Taking all into account, the correlation between mitochondrial dysfunction, chaperone/misfolded protein complex disruption, IR and brain impairment, emerge from these data. Therefore, the common features such as protein misfolding, aggregation, and IR are shared between AD and T2D (119), but more research is needed to tease out the importance and contribution of altered mitostasis in brain diseases. Though not in the brain but in white adipose tissue, rats under HFD have lower levels of Hsp60 and glucose intolerance (125), which may be a consequence of increased mitochondrial protein misfolding and thus dysfunction.

## The Special Case of Chaperones as Regulators of Central Insulin Sensitivity

### Importance of Insulin Sensitivity in the Brain and Consequences in Peripheral Tissues

Insulin signaling in the brain does not only regulate food intake and energy expenditure but also improves cognitive function (126, 127). Consistently, IR has been shown to contribute to hyperphagia and obesity (39) but as well as to comorbidities like neurodegenerative diseases and neurological alterations such as mood disorders (128, 129). In addition to insulin action on brain function, central insulin sensitivity impacts peripheral tissues and contributes to overall IR (41, 130, 131). Insulin in the brain suppresses liver glucose production partly through the stimulation of STAT3 tyrosine phosphorylation showing the tightly regulated brain-liver control (132). Hypothalamic insulin signaling is further needed for regulation of food intake and glucose uptake in peripheral organs as well as lipogenesis in adipose tissue *via* POMC insulin receptor action (133, 134). It has been shown that insulin regulates protein synthesis *via* the activation of mechanistic target of rapamycin (135). To ensure protein homeostasis and thus functional insulin signaling in the brain, a tightly regulated cluster of stress proteins like Hsps and proteases seems to be needed, which are part of the UPRmt. The protective role of Hsps mostly resides in their capacity to repair protein damage and denaturation (136, 137).

### Mitochondrial Chaperones As Regulators of Brain Insulin Sensitivity

The most prominent and well characterized stress proteins belong to the Hsp70 family with at least 11 different isoforms in the various compartments of the cell. The inducible Hsp70 is ubiquitously expressed and a highly conserved chaperone, localized in the cytosol and can be induced by heat, oxidative stress or by changes in the pH (138, 139). It has been shown that Hsp70 is a neuroprotective protein upregulated in the cortical brain of mice by alternate day fasting to ensure neuronal plasticity (139). Neuronal plasticity and neurogenesis can be negatively influenced by hippocampal IR (140) and so, neuronal insulin sensitivity is crucial for appropriate brain function. In addition, a recent study showed that intranasally administered Hsp70 improves insulin sensitivity in a diet-induced diabetic mouse model. This non-invasive procedure of administration of a highly abundant and easily producible therapeutic target like the chaperone Hsp70 could be a potential new treatment for diabetes (141). The mitochondrial member of the Hsp70 family, mtHsp70, encoded by the gene Hspa9 is also designated as mitochondrial chaperone glucose-regulated protein75 (Grp75) or mortalin. A study by Voloboueva et al. showed that overexpressing Grp75 in microglial BV-2 cells attenuated lipopolysaccharide-mediated inflammatory response and protects mitochondrial function (142). Strikingly, transient induction *via* heat shock as well as constitutively overexpressing hsp70F—a homolog of mtHsp70—in *C. elegans* extends life span (143). The extension of life span was also achieved in *Drosophila melanogaster* by the upregulation of Hsp70 via heat shock (144). Longevity is known to be associated with improved insulin signaling in humans (145). In addition, Grp75 is crucial for the formation of mitochondria-associated ER membranes (MAM) and altered MAM formation has been shown to induce IR (146). Protecting functional mitochondria is an important part of ensuring insulin sensitivity in the brain since mitochondrial dysfunction induces insulin and leptin resistance (7, 9, 147). Functional mitochondria rely on dynamic networks alternating between fusion and fission states. Under nutrient excess in HFD, these organelles undergo fission to form fragmented mitochondria to prevent excessive ATP synthesis (148). This process is mediated by DRP1 and its increased expression is sufficient to induce IR (43, 148). Additionally, the mitochondrial TRAP1—also called the mitochondrial Hsp90—plays an important role in the maintenance of mitostasis by using its antioxidant capacity as well as the regulation of mitophagy and mitochondrial dynamics (84–86). Reduced expression of TRAP1 has been linked to pathologies like PD and ischemic damage (Table 1). In contrast, TRAP1-KO in older mice leads to reduced weight and reduced glucose levels indicative of increased insulin sensitivity, which may be explained by increased fatty acid oxidation in these mice (87). Hence, changing mitochondrial chaperone activity and targeting mitochondrial dynamics is a promising therapeutic approach for increasing insulin sensitivity and controlling body weight.

A rather interesting mammalian copper chaperone, COX17p, is crucial for cytochrome c (COX) activity, however, not for other ETC complex subunits. Thus, in its absence, copper cannot be delivered to the mitochondria and this deficiency leads to disassembly of COX (149). Mutations in human COX17p lead to severe COX deficiency along with hypertrophic cardiomyopathy (102). COX deficiency is known to induce mitochondrial dysfunction and lead to accelerated apoptotic cell death by the upregulation of ceramide synthase 6 (CerS6) in response to oxidative stress (150). It has been shown that obesity is also able to induce CerS6 in adipose tissue of obese patients, which has been implicated in reduced insulin sensitivity and weight gain due to increased ceramide production (151). Whether this is might occur in brain remains unknown, but it indicates that a reduction of COX17p can induce brain IR. Taken together, it shows that regulating mitochondrial function can be a therapeutic approach of treating obesity-induced IR. We observed reduced expression of Hsp60 and 10 in T2D mice, which can cause mitochondrial dysfunction and hypothalamic IR [(7) and own observations]. Hsp60 and its co-chaperone Hsp10 are required for folding nuclear-encoded mitochondrial matrix proteins and are key players of the UPRmt by propagating ATP-dependent refolding of misfolded proteins. This mitochondria-to-nuclear signal transduction pathway can induce mitochondrial protective genes including the chaperones itself as well as mitochondrial proteases such as the LonP and the serine protease complex ClpP to restore cellular homeostasis (152). Mitochondrial protein aggregates activate c-Jun kinase (JNK) and its downstream transcription factor c-Jun (153), a kinase which induces IR (154). To highlight the importance of JNK-signaling for the development of IR, it has been shown that HFD-fed mice with inactivated JNK in the hypothalamus show overall improved insulin sensitivity in the brain as well as peripheral tissue (155).

Interestingly, apart from their classical function, mitochondrial chaperones can be found extracellularly in stressed condition and exert a pro-inflammatory response. Toll-like receptor 2 and 4 can be activated by host-derived Hsps including Hsp60 and Hsp70 resulting in increased cytokine production (156, 157). In the case of Hsp60, this can even lead to neurodegeneration via neuronal cell death and demyelination of the cerebral cortex as well as dopaminergic cell death during PD (158, 159). This shows that Hsps released from damaged cells are implemented in an inflammatory response and neuronal cell death. Taken together, both the reduction in the mitochondria as well as the release of Hsps can be detrimental to neurons.

In conclusion, functional mitochondria along with proper regulation of UPRmt are crucial for healthy brain function and metabolism and can ensure brain insulin sensitivity.

Many features of brain alterations observed during age-related neurodegenerative diseases are also observed in metabolic diseases pointing to common origins. In fact, some studies have shown that diabetic patients exhibit an increased risk of developing PD, AD, and IR (160–164). The common molecular underlying reason may evolve around mitochondrial dysfunction. The development and progression of PD and T2D shows mitochondrial dysfunction accompanied by an increase in oxidative stress and neuroinflammation (13). Since a hallmark of neurodegenerative diseases is the loss of (mito)proteostasis leading to the accumulation of aggregated proteins in the brain and in neurons (165), it is not surprising that its origin may underlie in mitochondrial dysfunction triggered by the disruption of the mitochaperone network followed by increased ROS formation which ultimately leads to protein carbonylation and aggregation. Along this line, aggregates are seen in most of neurodegenerative diseases, this includes the accumulation of Aβ and hyperphosphorylated tau in AD (166), the accumulation of synuclein in PD (167) or of the mutant form of the huntingtin protein in HD (168). Hyperphosphorylated tau has been also observed in brains of insulin receptor deficient mice (169).

Another type of mitochondrial dysfunction is shown by the disturbance of the mitophagy process which is found in PD. The accumulation of misfolded matrix proteins on healthy mitochondria induces PINK1/Parkin mediated mitophagy (30). This PINK1/Parkin complex seems to be disrupted leading to the accumulation of malfunctioning mitochondria and is linked to PD (170). Additionally, Parkin loss was also observed in brains of db/db and HFD mice (171).

But besides the impairment of protein degradation machineries by mutations, e.g., of LonP and ClpP (172), also the chaperone system can be compromised, as mutations of Hsp60 are implicated in hereditary spastic paraplegia (34). Furthermore, a discovered missense mutation of the Hspe1 gene (coding for Hsp10) has been associated with neurodegeneration (79) and thus likely to metabolic complications as well. Interestingly, reduction of these proteins also alter metabolism.

Moreover, in AD a downregulation of the Hsp70 family member, HspA9 was found (mtHsp70) (173). More evidence for this perspective originates from Hsp60 inhibition which results in Aβ-induced disturbance of complex IV assembly and therefore, indicating a role in the mitochondrial stress response against the proteotoxic stress in AD (174).

A mutation of the HtrA2 gene is associated with Parkinson’s disease (175). Interestingly, a knockout mutation of the Htra2/Omi gene in mouse leads to neuromuscular changes similar to PD (176), while cellular studies revealed that HtrA2 knockout leads to mitochondrial dysfunction, accumulation of unfolded proteins, enhanced CHOP expression and increased ROS production (177), features also seen in metabolic disorders. In HD, mitochondrial bioenergetics and dynamics are affected by the pathological state, mitochondrial phenotypes which have been observed in brains of obese mice. However, to which extend mitochondrial chaperones are affected under this disease remains unclear and requires further investigation.

## Conclusion

Mitochondrial chaperones are crucial players for mitochondrial protein synthesis, protein folding, energy production, regulation of ROS and ion homeostasis (Figure 1). Age-related neurodegenerative and metabolic diseases show defects on mitostasis and chaperone expression/activity, which points to a common molecular origin.

In this perspective paper, we provided evidence that brain mitochondrial dysfunction is a likely result from the disruption of the mitochaperone network affecting brain insulin sensitivity. This shift in brain metabolism contributes to the development of aging, age-related and metabolic diseases.

We therefore postulate that a dysregulation of the mitochaperone network directly affects brain function and insulin sensitivity, acting as a percursor event for the development of neurodegenerative and especially metabolic diseases. To gain more insights into this complex interaction, more research needs to focus on the interplay of mitochondrial chaperone activity in the brain and subsequent alterations in brain function and metabolism, in order to fully understand the process between mitochaperone disruption in the brain and the development of metabolic diseases.

In conclusion, supporting mitochaperone function in metabolic and neurodegenerative diseases can improve brain insulin signaling as well as metabolism and represents a novel strategy to improve neuronal health and metabolism contributing to healthy aging.

## Author Contributions

JC and AK formulated the hypothesis and structure. JC, KW, TG, and AK wrote the manuscript. JC and AK prepared the figures. KW prepared the table. JC, KW, and AK revised and proofread the manuscript.

## Conflict of Interest Statement

The authors declare that the research was conducted in the absence of any commercial or financial relationships that could be construed as a potential conflict of interest.
